# Alzheimer’s disease pathology and shunt surgery outcome in normal pressure hydrocephalus

**DOI:** 10.1371/journal.pone.0182288

**Published:** 2017-08-07

**Authors:** Sevil Yasar, Ignacio Jusue-Torres, Jennifer Lu, Jamie Robison, Mira A. Patel, Barbara Crain, Kathryn A. Carson, Jamie Hoffberger, Sachin Batra, Eric Sankey, Abhay Moghekar, Daniele Rigamonti

**Affiliations:** 1 Department of Medicine, Johns Hopkins School of Medicine, Baltimore, Maryland, United States of America; 2 Department of Neurology, Johns Hopkins School of Medicine, Baltimore, Maryland, United States of America; 3 Department of Neurosurgery, Loyolla University Chicago Health Sciences Division, Maywood, Illinois, United States of America; 4 Albany Medical College, Albany, New York, United States of America; 5 Department of Neurosurgery, Johns Hopkins School of Medicine, Baltimore, Maryland, United States of America; 6 Department Otolaryngology, Washington University School of Medicine, St. Louis, Missouri, United States of America; 7 Department of Pathology, Johns Hopkins School of Medicine, Baltimore, Maryland, United States of America; 8 Department of Epidemiology, Johns Hopkins Bloomberg School of Public Health, Baltimore, Maryland, United States of America; 9 Department of Surgery, Harvard Medical School, Brigham and Women Hospital, Boston, Massachusetts, United States of America; 10 Department of Neurosurgery, Duke University Medical Center, Durham, North Carolina, United States of America; Biomedical Research Foundation, UNITED STATES

## Abstract

We aimed to determine whether presence of AD neuropathology predicted cognitive, gait and balance measures in patients with idiopathic normal pressure hydrocephalus (iNPH) after shunt surgery. This is a prospective study of gait and balance measured by Timed Up and Go (TUG) and Tinetti tests, and cognitive function measured by Mini Mental Status Exam (MMSE), before and after shunt surgery in participants 65 years and older with iNPH at the Johns Hopkins University. Random effects models were used and adjusted for confounders. 88 participants were included in the analysis with a median (IQR) time of 104 (57–213) days between surgery and follow-up. 23 (25%) participants had neuritic plaques present (NP+) and were significantly older [76.4 (6.0) years], but were otherwise similar in all demographics and outcome measures, when compared to the group without neuritic plaques (NP-). NP- and NP+ participants equally improved on measures of TUG (β = -3.27, 95% CI -6.24, -0.30, p = 0.03; β = -2.37, 95% CI -3.90, -0.86, p = 0.02, respectively), Tinetti-total (β = 1.95, 95% CI 1.11, 2.78, p<0.001; β = 1.72, 95% CI 0.90, 2.53, p<0.001, respectively), -balance (β = 0.81, 95% CI 0.23, 1.38, p = 0.006; β = 0.87, 95% CI 0.40, 1.34, p<0.001, respectively) and -gait (β = 1.03, 95% CI 0.61, 1.45, p<0.001; β = 0.84, 95% CI 0.16, 1.53, p = 0.02, respectively), while neither NP- nor NP+ showed significant improvement on MMSE (β = 0.10, 95% CI -0.27, 0.46, p = 0.61, β = 0.41, 95% CI -0.27, 1.09, p = 0.24, respectively). **In summary**, 26% of participants with iNPH had coexisting AD pathology, which does not significantly influence the clinical response to shunt surgery.

## Introduction

Idiopathic normal pressure hydrocephalus (iNPH) is a clinical syndrome characterized by insidious onset of gait, cognitive and urinary dysfunction [1]. iNPH is mainly a disease of the elderly with an increasing prevalence associated with age, 0.2% between age 70–79 years and 5.9% over age 80 years, although data also suggest that it is extremely under-diagnosed [2]. iNPH diagnosis is based on clinical presentation, neuroimaging and response to cerebrospinal fluid (CSF) drainage and is often a diagnosis of exclusion [3–6]. It is treated with shunt placement to divert CSF flow with clinical improvement in 24–80% of the cases immediately after shunt surgery and 50% after 3-year follow-up [7]. The wide range of effectiveness may be partially due to the presence of comorbid neurodegenerative and/or cerebrovascular diseases. The inadequate sensitivity and specificity of tests assessing shunt-responsiveness in the presence of any of these comorbidities may complicate the interpretation of long-term clinical outcomes of shunt placement [8–10].

Alzheimer’s disease (AD) is the most common neurodegenerative disease in older people [11] and is a common comorbid condition of iNPH ranging between 18–42% [12–16], with overlapping clinical features with iNPH. Efforts have been made to clinically differentiate iNPH and AD in order to effectively guide therapy. Studies have shown that clinical indicators that may distinguish iNPH-dominant vs. AD-dominant disease process include gait problems as the presenting symptom in iNPH and cognitive problems presenting in AD [17].

Studies evaluating the effect of shunt placement in people with iNPH alone or with comorbid AD have shown equivocal results. Some showing similar improvement in both groups, others showing improvement in gait only in participants with comorbid AD pathology found on brain biopsy obtained during shunt surgery [12,14,16–20]. Additionally, a study of 39 participants using phosphorylated tau and amyloid beta 1–42 ratio as measure of AD pathology in ventricular CSF showed less improvement in gait and cognition among participants with high ratio [21], while another small study of 10 participants using PET amyloid beta (Aβ) imaging showed less cognitive improvement in participants with high Aβ [22].

The aim of this study was to determine in a larger sample whether the presence of AD neuropathology, neuritic plaques (NP) and neurofibrillary tangles (NFT), predicted change in objectively measured cognitive, gait and balance measures in patients with idiopathic normal pressure hydrocephalus (iNPH) after CSF drainage trial and after shunt surgery.

## Materials and methods

### Participants

Study participants were recruited from the Johns Hopkins Cerebrospinal Fluid Disorder Program within the department of Neurology and Neurosurgery. Demographic and baseline clinical characteristics including age, race, gender, years of education, smoking history, and medical history which included history of hypertension, diabetes mellitus, hyperlipidemia, coronary artery disease, stroke, transient ischemic attack, Parkinson’s disease, spinal disorder, peripheral neuropathy, cerebellar ataxia, osteoarthritis, and dementia were collected. The study was approved by the Johns Hopkins Institutional Review Board Committee and participants provided written informed consent before participation for brain biopsy in conjunction with shunt insertion surgery.

Ninety-eight patients were seen in the center between 2009 and 2013 for evaluation after meeting criteria for probable iNPH, including symptoms, signs, MRI findings of ventriculomegaly with Evans index > 0.3 and open cerebral aqueduct, and normal opening pressure according to international guidelines [23]. Patients were selected for shunt surgery based on initial evaluation and diagnosis of iNPH and significant improvement on the Tinetti-total, -gait and—balance scale [24] and the Timed-Up and Go (TUG) task [25], after either an outpatient large volume lumbar drainage trial with removal of 30–40 cc of CSF or by a more extended inpatient drainage trial of a total of 300–600 cc of CSF using international guidelines [26].

Of the 98 participants, 10 participants were excluded from the analysis because there was no follow-up testing done within 1 year.

### Neuropathologic examination

A cortical biopsy was taken from the catheter insertion site in the right parietal cortex. The tissue was fixed in neutral buffered formalin and embedded in paraffin. Sections were stained with hematoxylin and eosin using Hirano silver method (modified Bielschowsky stain) [27], and immunohistochemistry for phosphorylated tau (AT8, Research Diagnostic, Inc., Planders, NJ), and for amyloid-beta (6F/3D, DAKO, Carpinteria, CA) was performed. The Consortium to Establish a Registry of Alzheimer Disease (CERAD) criteria [28] was used to measure neuritic plaque count in the 1-sq-mm area of the brain regions provided. The neuritic plaque (NP) count was rated as none (C0), sparse (C1), moderate (C2), or frequent (C3). Neurofibrillary tangles (NFT) were assessed and were identified in either the silver stain or the tau immunostain.

### Outcome measures

Gait testing results, including Tinetti and TUG were collected before and after a large volume ELD trial and at least 3–6 months after shunt surgery. Similarly, cognitive test results using the Mini Mental Status Exam (MMSE) [29] were collected before and after shunt surgery. Presence of dementia was determined at baseline by the examining and/or reviewing physician using the DSM-IV criteria [30]. Whether the participant developed gait problems prior to cognition was also recorded.

### Statistical methods

Patient baseline characteristics overall and by NP category were summarized using frequencies with percentages or means with standard deviations (SDs). Participants with neuritic plaques (NP+) were compared to those without neuritic plaques (NP-) using Kruskal-Wallis test for equal medians or analysis of variance test of equal means for continuous measures and Fisher’s exact test for categorical measures.

For each outcome measure, stratified by NP absence or presence, a linear random effects model with a random intercept and random time slope was used to simultaneously model baseline test scores and rate of change over time. We used linear random effects model in order to account for within subject correlation, which allows each participant to have a different starting point; as well as a subject specific slope, allowing each participant to have a different rate of change. Additionally, the random effects model has the ability to handle unequal lengths of follow-up and data missing at random.

Analyses in Model 1 were unadjusted and in Model 2 adjusted for potential confounding. For cognition models were adjusted for age, gender, race, education, smoking history, dementia diagnosis at baseline, time between surgery and follow-up and a variable of “number of additional diseases” was used to reduce the number of confounders, after we separately assessed individual diseases and found no significant associations between presence or absence of NP and history of hypertension, diabetes mellitus, hyperlipidemia, coronary artery disease, stroke, transient ischemic attack. While for gait models were adjusted for age, time between surgery and follow-up, and a variable of “number of additional diseases” including stroke, transient ischemic attack, Parkinson’s disease, spinal disorder, peripheral neuropathy, cerebellar ataxia, and osteoarthritis.

Additionally, we have used ANOVA in order to test whether the change from pre LP and post surgery between NP groups differs.

Analyses were performed using STATA version 12.1 (Stata Corp LP, Inc., College Station, TX). All reported *p* values are two-sided and significance was set at p<0.05.

## Results and discussion

### Participants

There were a total of 88 participants with mean age of 71.7 (8.0) years, 52 (59%) were males, 80 (91%) were white, and the mean years of education was 15.2 (2.9) (Table 1). Of the 88 participants 23 (26%) had sparse or moderate amount of NP present (NP+), while 65 (74%) had NP absent (NP-). NP+ participants were significantly older 76.4 (6.0) years of age when compared to NP- participants 70.1 (7.9) years (p<0.001). None of the NP- participants had NFT and only 6 (26%) NP+ participants had NFT present. Sixty-two (70%) participants received ventriculoperitoneal shunts and 26 (30%) ventriculoatrial shunts. Both groups were otherwise similar on all demographic measures “Table 1”.

**Table 1:** Baseline characteristics of study participants.

	All Participants N = 88	NP-^#^ N = 65	NP+^§^ N = 23	*p*-value
Age at surgery, Mean (SD), years	71.7 (±8.0)	70.1 (±7.9)	76.4 (±6.0)	<0.001
Gender, Male, N(%)	52 (59)	36 (55)	16 (70)	0.32
Race, White, N (%)	80 (91)	59 (91)	21 (92)	0.36
Education, Mean (SD), years	15.2 (±2.9)	15.0 (±2.9)	15.9 (±3.1)	0.20
Hypertension, N(%)	56 (64)	38 (58)	18 (78)	0.13
Diabetes mellitus, N(%)	29 (33)	21 (32)	8 (35)	0.99
Hyperlipidemia, N(%)	48 (54)	33 (51)	15 (65)	0.33
Coronary artery disease, N(%)	19 (21)	14 (21)	5 (22)	0.99
Stroke/TIA^¶^, N(%)	14 (16)	11 (17)	3 (13)	0.99
Parkinson’s disease, N(%)	4 (4)	3 (5)	1 (4)	0.99
Spine disease, N(%)	18 (20)	12 (18)	6 (26)	0.54
Neuropathy, N(%)	18 (20)	14 (21)	4 (17)	0.77
Osteoarthritis, N(%)	34 (39)	26 (0)	8 (35)	0.80
Ventriculoperitoneal shunt, N(%)	62 (70)	43 (66)	19 (83)	
Ventriculatrial shunt, N(%)	26 (30)	22 (44)	4 (17)	0.33
Time between surgery and first follow-up visit, median (IQR), days	104 (57–213)	101.5 (50–230)	104 (64–159)	0.88
Neurofibrillary tangles, N(%)	6 (7)	0 (0)	6 (26)	<0.001

^#^NP- Neuritic plaques absent.

^§^NP+ Neuritic plaques present.

^¶^Transient Ischemic Attack (TIA).

At baseline the mean (SD) MMSE score was 27 (2.7), TUG time was 22.1 (16.2) seconds and Tinetti-total score was 19.6 (5.9) for all participants, and there was no difference between NP+ and NP- participants (Table 2). Similarly, no significant difference was found in outcome measures of MMSE, TUG, Tinetti-total, Tinetti-balance and Tinetti-gait scores between NP+ and NP- participants after ELD trial and surgery “Table 2”.

**Table 2:** Gait and cognitive measures before and after large volume ELD by neuritic plaque status.

	All Participants N = 88	NP-^#^ N = 65	NP+^§^ N = 23	*p*-value
**A) Before ELD**^¶^ **trial**				
MMSE^♮^, Mean(SD)	27.0 (±2.7)	27.0 (±2.8)	27.1 (±2.9)	0.93
TUG*, Mean(SD), seconds	22.1 (±16.2)	21.8 (±15.9)	23.0 (±17.4)	0.78
Tinetti Total, Mean (SD)	19.6 (±5.9)	19.6 (±6.2)	19.4 (±5.1)	0.87
Tinetti Balance, Mean (SD)	11.2 (±3.5)	11.3 (±3.7)	11.1 (±3.2)	0.81
Tinetti Gait, Mean (SD)	8.2 (±3.1)	8.2 (±3.2)	8.3 (±2.7)	0.89
**B) After ELD**^¶^ **trial**				
MMSE, Mean(SD)	-	-	-	-
TUG*, Mean(SD), seconds	15.2 (±7.7)	15.2 (±7.2)	15.4 (±9.1)	0.91
Tinetti Total, Mean (SD)	23.0 (±4.9)	23.1 (+5.0)	22.7 (±5.0)	0.65
Tinetti Balance, Mean (SD)	12.9 (±3.1)	13.1 (+3.0)	12.4 (±3.2)	0.34
Tinetti Gait, Mean (SD)	10.0 (±2.3)	10.0 (+2.3)	10.2 (±2.3)	0.71
**C) After shunt surgery**				
MMSE, Mean(SD)	27.2 (±2.9)	27.1 (±3.1)	27.5 (±2.4)	0.62
TUG, Mean(SD), seconds	13.7 (±12.9)	13.8 (±12.2)	13.4 (±3.5)	0.89
Tinetti Total, Mean (SD)	23.8 (±5.6)	24.0 (±5.8)	23.1 (±5.3)	0.55
Tinetti Balance, Mean (SD)	13.3 (±3.3)	13.3 (±3.6)	13.4 (±3.6)	0.92
Tinetti Gait, Mean (SD)	10.6 (±2.4)	10.6 (±2.6)	10.5 (±1.8)	0.80

^#^NP- Neuritic plaques absent.

^§^NP+ Neuritic plaques present.

^¶^ELD—Extended lumbar drainage.

^♮^MMSE—Mini Mental Status Exam.

*TUG—Timed up and go test.

### Outcomes for total sample, and after stratification by presence or absence of neuritic plaques

#### Before and after large volume CSF ELD trial

All participants showed significant improvement in measures of TUG (β = -5.97, 95% CI -8.25, -3.70, p<0.001), Tinetti-total (β = 3.17, 95% CI 2.39, 3.95, p<0.001), Tinetti-balance (β = 1.57, 95% CI 0.98, 2.16, p<0.001) and -gait (β = 1.59, 95% CI 1.08, 2.09, p<0.001) (Table 3). After stratifying by NP status, both NP- and NP+ participants showed significant improvement in measurements of TUG (β = -6.05, 95% CI -8.93, -3.17, p<0.001; β = -5.59, 95% CI -8.36, -2.81, p<0.001, respectively), Tinetti-total (β = 2.91, 95% CI 2.04, 3.78 p<0.001; β = 3.93, 95% CI 2.16, 5.70, p<0.001, respectively), -balance (β = 1.41, 95% CI 0.72, 2.09, p<0.001; β = 1.91, 95% CI 0.60, 3.23, p = 0.004, respectively) and -gait score (β = 1.47, 95% CI 0.90, 2.05, p<0.001; β = 1.91, 95% CI 0.85, 2.96, p<0.001, respectively) “Table 3”.

**Table 3:** Random effects analysis for gait scores before and after large volume ELD trial by neuritic plaque status.

	Unadjusted Model		Adjusted Model^¶^	
	β-coefficient (95% CI)	*p*-value	β-coefficient (95% CI)	*p*-value
TUG All	-7.10 (-9.36, -4.84)	<0.001	-5.97 (-8.25, -3.70)	<0.001
TUG NP-^#^	-6.77(-9.49, -4.06)	<0.001	-6.05 (-8.93, -3.17)	<0.001
TUG NP+^§^	-8.01(-12.09, -3.92)	<0.001	-5.59 (-8.36, -2.81)	<0.001
Tinetti Total All	3.29 (2.58, 4.00)	<0.001	-3.17 (2.39, 3.95)	<0.001
Tinetti Total NP-	3.22 (2.38, 4.07)	<0.001	2.91 (2.04, 3.78)	<0.001
Tinetti Total NP+	3.45 (2.03, 4.86)	<0.001	3.93 (2.16, 5.70)	<0.001
Tinetti Balance All	1.60 (1.08, 2.12)	<0.001	1.57 (0.98, 2.16)	<0.001
Tinetti Balance NP-	1.65 (1.02, 2.28)	<0.001	1.41 (0.72, 2.09)	<0.001
Tinetti Balance NP+	1.46 (0.46, 2.46)	0.004	1.91 (0.60, 3.23)	0.004
Tinetti Gait All	1.69 (1.24, 2.14)	<0.001	1.59 (1.08, 2.09)	<0.001
Tinetti Gait NP-	1.57 (1.03, 2.11)	<0.001	1.47 (0.90, 2.05)	<0.001
Tinetti-Gait NP+	1.96 (1.11, 2.81)	<0.001	1.91 (0.85, 2.96)	<0.001

^#^NP- Neuritic plaques absent.

^§^NP+ Neuritic plaques present.

^¶^Models were adjusted for: age, gender, education, smoking history, presence of dementia, gait before cognitive symptoms, and composite number of additional diseases.

#### Before large volume CSF ELD trial and after shunt surgery

All participants showed significant improvement in measures of TUG (β = -3.18, 95% CI -5.41, -0.94 p = 0.005), Tinetti-total (β = 1.92, 95% CI 1.26, 2.58, p<0.001), -balance (β = 0.86, 95% CI 0.42, 1.29, p = 0.006) and -gait score (β = 1.00, 95% CI 0.64, 1.36, p<0.001), while participants did not improve significantly on cognitive measure of MMSE (β = 0.15, 95% CI -0.17, 0.47, p = 0.37) (Table 4). After stratifying for NP status, both NP- and NP+ participants showed significant improvement in measurements of TUG (β = -3.27, 95% CI -6.24, -0.30, p = 0.03; β = -2.37, 95% CI (-3.90, -0.86, p = 0.02, respectively), Tinetti-total (β = 1.95, 95% CI 1.11, 2.78, p<0.001; β = 1.72, 95% CI 0.90, 2.53, p<0.001, respectively), -balance (β = 0.81, 95% CI 0.23, 1.38, p = 0.006; β = 0.87, 95% CI 0.40, 1.34, p<0.001, respectively) and -gait score (β = 1.03, 95% CI 0.61, 1.45, p<0.001; β = 0.84, 95 CI 0.16, 1.53, p = 0.02, respectively), while neither NP- nor NP+ showed significant improvement on cognitive measure of MMSE (β = 0.10, 95% CI (-0.27, 0.46, p = 0.61; β = 0.41, 95% CI -0.27, 1.09, p = 0.24, respectively) “Table 4”.

**Table 4:** Random effects for cognitive and gait scores before large volume ELD trial and after surgery by neuritic plaque status.

	Unadjusted Model		Adjusted Model^¶^	
	β-coefficient (95% CI)	*p*-value	β-coefficient (95% CI)	
MMSE All	0.15 (-0.16, 0.45)	0.34	0.15 (-0.17, 0.47)	0.37
MMSE NP-^#^	0.13 (-0.20, 0.47)	0.44	0.10 (-0.27, 0.46)	0.61
MMSE NP+^§^	0.21 (-0.46, 0.88)	0.54	0.41 (-0.27, 1.09)	0.24
TUG All	4.22 (-6.38, -2.05)	<0.001	-3.18 (-5.41, -0.94)	0.005
TUG NP-	-4.00 (-6.78, -1.22)	0.005	-3.27 (-6.24, -0.30)	0.03
TUG NP+	-4.76 (-7.93, -1.58)	0.003	-2.37 (-3.90, -0.86)	0.02
Tinetti-Total All	2.08 (1.51, 2.66)	<0.001	1.92 (1.26, 2.58)	<0.001
Tinetti Total NP-	2.19 (1.43, 2.95)	<0.001	1.95 (1.11, 2.78)	<0.001
Tinetti Total NP+	1.79 (1.16, 2.42)	<0.001	1.72 (0.90, 2.53)	<0.001
Tinetti Balance All	0.99 (0.61, 1.38)	<0.001	0.86 (0.42, 1.29)	<0.001
TinettiBalance NP-	0.99 (0.47, 1.15)	<0.001	0.81 (0.23, 1.38)	0.006
Tinetti Balance NP+	0.94 (0.48, 1.39)	<0.001	0.87 (0.40, 1.34)	<0.001
Tinetti Gait All	1.09 (0.77, 1.40)	<0.001	1.00 (0.64, 1.36)	<0.001
Tinetti Gait NP-	1.11 (0.73, 1.49)	<0.001	1.03 (0.61, 1.45)	<0.001
Tinetti Gait NP+	1.04 (0.48, 1.61)	<0.001	0.84 (0.16, 1.53)	0.02

^#^NP- Neuritic plaques absent.

^§^NP+ Neuritic plaques present.

^¶^Models were adjusted for: age, gender, education, smoking history, presence of dementia, gait before cognitive symptoms, and composite number of additional diseases.

The MMSE improved by 0.37 points in NP- group and by 0.44 points in NP+ group, while TUG improved by 7.9 seconds in NP- group and by 9.3 seconds in NP+ group, and the improvements were similar between the groups (p = 0.93 and p = 0.78, respectively). Similarly, total Tinetti scores improvement by 4.4 points in NP- group and by 3.5 points in NP+ group and the improvements did not differ between groups (p = 0.52).

#### Clinical outcome differences by plaque severity

The study sample of NP+ participants was further divided into categories based on CERAD criteria of neuritic plaques being sparse (C1) (N = 16, 18%) or moderate (C2) (N = 7, 8%) The only significant differences in baseline characteristics between those with absent, sparse or moderate plaques were age at surgery 70.1 (7.9), 75.1 (5.5) and 79.4 (6.5) years (p = 0.001). Only six participants had NFT present with two (12%) among participants with sparse and four (57%), p<0.001. There were no significant differences between those participants with absent, sparse or moderate plaques on MMSE, TUG, Tinetti-total, -balance, -gait before and after the ELD trial or before the ELD trial and after shuntings. Sample sizes for the three groups were too small to perform regression analysis comparing them.

## Conclusions

iNPH is a distinct cause of gait and cognitive impairment in older populations and it is reversible by shunt surgery. The relevance of comorbid conditions in iNPH to shunt-responsiveness is of critical importance to prevent unnecessary surgical procedures in patients that may derive little or no benefit. Previous studies have explored the association between comorbid AD pathology with iNPH and shunt-responsiveness, and results are equivocal. However in some studies AD pathology severity precluded clinical improvement [16–18,20]. The different findings could be partially explained by methodological issues such as different sample size, frequently small sample size [12,14,16,18], different methods used to measure Aβ in brain by means of biopsy or PET [22] or CSF [31], and application of different outcome measures such as subjective report [20].

In this larger prospective study we sought to clarify this debate by evaluating associations between biopsy-confirmed AD neuropathology and objective measures of cognition, gait and balance after ELD trial and shunt surgery in 88 community-dwelling older participants diagnosed with iNPH and treated with shunt placement.

We have found that 26% of participants with iNPH had coexisting AD pathology, which is similar to previous studies [12,20,32]. The discrepancy between our findings and another biopsy study showing a 46% prevalence of AD pathology [33] could be a result of biopsy samples being obtained from different areas, in our case from parietal lobe. Additionally, our findings also differ from Aβ PET imaging study showing elevated Aβ in 50% of participants with iNPH [22], an autopsy study showing 56% comorbid AD pathology when obtained from multiple brain areas [34] and another study showing 68% of comorbid AD measured by phosphorylated tau and amyloid beta ratio in ventricular CSF [21], which all could be a results of biopsy samples being obtained during shunt surgery only from one area, thus resulting possibly in under diagnosis. The high prevalence of comorbid AD pathology in iNPH raises the possibility of a common pathway. Accumulation of Aβ in the meninges of AD may cause resistance to CSF outflow and lead to elevated CSF pressures, while increases in CSF pressure in iNPH may lead to decreased production of CSF and declining Aβ clearance, resulting in AD pathology [35]. Regardless of the directionality of dysfunction, it is clear that iNPH and AD may be related through CSF circulatory pathology.

Participants with and without NP showed similar significant improvement in gait and balance after both ELD trial and shunt surgery, while no significant improvement in cognition was seen after shunt surgery. There has been only one recent study [20] that has also included large volume preoperative CSF ELD trial to predict shunt response and found difference to ELD response, but not to shunt response, when stratified by AD pathology. The difference between results could be explained by the longer follow-up time, which was 35 months, different gait and cognitive measures, and different statistical analysis used.

In our study there was no significant improvement on MMSE, which was independent of the NP status. This could be explained by mild disease burden since none of the participants had severe and only 7 had moderate plaque burden, which was also reflected by high baseline MMSE score of 27. This is supported by an observational study by Hashimoto et al. [36] where baseline MMSE was 23, by clinical trial by Klinge et al. [37] where baseline MMSE was 24 and by SINPHONI-2 clinical trial [31] where baseline MMSE was 20. However in a previous study where participants had low MMSE of 18 at baseline there was also no improvement in cognition after shunt placement [14]. This raises the possibility of MMSE not being a sensitive measure and perhaps more detailed cognitive testing should be performed similar to a small study of 37 participants [18] and another clinical trial of 68 participants [38] where a larger neuropsychological battery was able to capture differences. Additionally, the relatively short follow-up time (104 days) may not be sufficient to capture changes in clinical outcome measures.

In our study we have found similar and significant improvement in measures of gait and balance in both NP groups similar to two other studies [14,20] while Hamilton and colleagues found significant improvement in gait and balance only in participants with no AD pathology, and this improvement decreased with increasing AD pathology severity [18].

There were a number of advantages of this study. First, our study included a larger well-characterized cohort, with detailed evaluation, testing and medical history, who underwent rigorous evaluation for shunt eligibility. Additionally, we had objective cognitive, gait and balance measures both before and after surgery.

This study also had limitations. The sample size was small and did not allow us to assess clinical outcome by NP severity. Our study population was highly educated and homogenous with respect to race, limiting generalizability. Additionally, we were unable to account for effects of prior history and other causes for dementia. As in all observational studies, our results may also be vulnerable to confounding, which we sought to address by adjusting for history of coexisting diseases affecting gait and cognition.

In summary, iNPH is a disabling disease of the elderly that significantly reduces quality of life for which treatment is available in the form of shunt placement. Our study suggests that the presence of mainly mild AD pathology, specifically amyloid beta, on cortical biopsy had no effect on both gait and cognitive outcomes after ELD trial and after shunt surgery compared to those individuals with no evidence of AD pathology. Further larger studies with brain biopsy evaluating for both amyloid plaque and neurofibrillary tangles, detailed cognitive and gait testing are needed in order to better address the question of the role of coexisting AD pathology on shunting outcome in iNPH patients.
